# Effect of Surgical Specialty on Postoperative Complications of Rhinoplasty

**DOI:** 10.1002/ohn.1338

**Published:** 2025-07-17

**Authors:** Sree Chinta, Keshav D. Kumar, Dhiraj Sibala, Afash Haleem, Akshay K. Warrier, Joseph A. Celidonio, Jean Anderson Eloy

**Affiliations:** ^1^ Department of Otolaryngology–Head and Neck Surgery Rutgers New Jersey Medical School Newark New Jersey USA; ^2^ Rutgers New Jersey Medical School, Center for Skull Base and Pituitary Surgery Neurological Institute of New Jersey Newark New Jersey USA

**Keywords:** head and neck surgery, plastic surgery, rhinoplasty

## Abstract

**Objective:**

Rhinoplasty is a cosmetic procedure that is typically performed by either otolaryngologists (ear, nose, and throat [ENT]) or plastic surgeons. This study specifically investigates the association between surgical specialty and complications in rhinoplasty.

**Study Design:**

Retrospective database review.

**Setting:**

US hospitals.

**Methods:**

The National Surgical Quality Improvement Program database was queried for patients who underwent rhinoplasty between 2005 and 2020. Univariate and multivariable analyses were conducted to determine associations between surgical specialty and postoperative complications.

**Results:**

In total, 2127 patients undergoing rhinoplasty were queried. Of the cohort, 1253 (58.9%) were operated on by ENT and 874 (41.1%) were operated on by plastic surgeons. ENT patients were more likely to be male (55.8% vs 46.7%, *P* < .001), have a higher American Society of Anesthesiologists (ASA) classification (ASA class I: 13.0% vs 19.2%, *P* < .001), be smokers (19.2% vs 14.4%, *P* = .002), and have prolonged operative time (59.9% vs 44.4%, *P* < .001). Univariate analysis also showed a significantly lower incidence of sepsis (0.1% vs 0.8%, *P* = .008) among ENT patients. Multivariable analyses revealed that plastics patients had greater odds of postoperative sepsis (odds ratio: 23.716, CI: 2.025‐277.764, *P* = .012).

**Conclusion:**

Surgical specialty has been shown to impact outcomes of other head and neck procedures. This study suggests an independent association between surgical specialty and 30‐day postoperative sepsis in rhinoplasty.

**Level of Evidence:**

IV.

Rhinoplasty is the most common facial cosmetic surgical procedure performed in the United States, with more than 2 million cases performed annually for both functional and esthetic purposes.^1^ There is an overlap between the surgical procedures, especially in the domain of facial plastics and reconstructive surgery, performed by otolaryngologists and plastic surgeons given that plastic surgeons are not restricted to any single anatomical region.^2^ Both otolaryngologists and plastic surgeons perform functional rhinoplasty for oncologic reconstruction, facial trauma, congenital defects, and nasal airflow obstruction.^3^ Although rhinoplasty is generally a safe, outpatient procedure, up to 15% of patients have subsequent revision rhinoplasties, and 3% have complications such as epistaxis, septal hematoma, cellulitis, abscesses, septal perforation, and adverse esthetic outcomes.^4^, ^5^, ^6^

The direct relationship between demographic and comorbidity variables such as male sex, older age, bleeding disorders, and higher American Society of Anesthesiologists (ASA) class with postoperative complications in rhinoplasty cases in addition to the inverse relationship between surgeon volume and adverse outcomes in otolaryngology procedures has been well‐established.^7^, ^8^, ^9^ However, there is a scarcity of well‐designed studies assessing the differences in postoperative complications in patients undergoing surgery when performed by otolaryngologists versus plastic surgeons. The few prior studies that have been conducted on this topic have found no differences in the rate of reoperations, unplanned readmissions, or mortality between otolaryngologists and plastic surgeons who perform rhinoplasty for cleft repairs or pediatric rhinoplasty in general.^10^, ^11^

There has been a decreasing proportion of rhinoplasties performed by plastic surgeons relative to those performed by otolaryngologists in recent years.^12^ Furthermore, there is a lack of patient awareness of the differences between facial plastic and reconstructive surgeons with otolaryngology residency training and surgeons who have plastic surgery residency training.^13^ The changing market share of rhinoplasties in conjunction with the lack of choice or awareness of the specialty of surgeon among patients due to emergent indication, geographic limitations, or surgeon availability highlights the importance of studying differences in postoperative outcomes by the specialty of the surgeon.

However, the relationship between surgical specialty and outcomes in adult rhinoplasty in the hospital setting has not been studied. This study aims to utilize a national database to characterize the differences in 30‐day postoperative outcomes between patients who were operated on by plastic surgeons compared to those who were operated on by otolaryngologists.

## Methods

### Data Source

The American College of Surgeons National Surgical Quality Improvement Program (ACS‐NSQIP) is a data registry published annually by the ACS that includes patient surgical cases and their associated demographics, comorbidities, and postoperative data. Data are collected from more than 700 hospitals nationwide and include more than 1 million cases annually. It consists of nationally validated, risk‐adjusted data and has been used extensively to assess outcomes of numerous surgical procedures. The data that are entered into the database by Surgical Clinical Reviewers at each of the member institutions do not contain any patient identifiers.

### Patient Selection

Surgical cases from the years 2005 to 2020 were included in this study, filtered using primary, other, and concurrent procedures for Current Procedural Terminology (CPT) codes related to rhinoplasty.^7^ The CPT codes used in this study included those for primary, complete external rhinoplasty (30410), primary rhinoplasty with major septal repair (30420), minor revision rhinoplasty (30430), secondary rhinoplasty with intermediate revision and bony work with osteotomies (30435), and secondary rhinoplasty with major revision including nasal tip adjustment and osteotomies (30450), consistent with previous retrospective database studies focused on patients undergoing rhinoplasty.^14^, ^15^ Patients with missing demographic and comorbidity variables were excluded from the study. The NSQIP database does not include any pediatric patients, so all queried patients were aged 18 years and older. As NSQIP consists of deidentified patient data, this study was exempted from the Rutgers New Jersey Medical School Institutional Review Board.

### Variable Selection

Demographic data, including age, sex, race, and American Society of Anesthesiologists (ASA) class, were collected for each case. Comorbidity data were also collected including smoking status, diabetes (requiring treatment with oral agents or insulin), dyspnea (dichotomized with presence consisting of dyspnea at rest or with moderate exertion), chronic obstructive pulmonary disease (COPD), hypertension requiring medication, open wound with or without infection, bleeding disorders, obesity (body mass index greater than or equal to 30), steroid or immunosuppressant use for a chronic condition, on dialysis preoperatively, presence of disseminated cancer, history of congestive heart failure, and weight loss of greater than 10% of body weight over the preceding 6 months. Prolonged operative time was defined as cases that were longer in duration than the 50th percentile for operative length for all rhinoplasty cases queried in this study (155 minutes). The independent variable was the surgical specialty of the surgeon, and cases were divided into two groups based on the specialty of the operating surgeon: ear, nose, and throat (ENT) and plastics. Cumulative wound complications included superficial surgical site infections, deep surgical site infections, organ/space surgical site infections, and wound dehiscence. Medical complications assessed included pneumonia, on a ventilator for greater than 48 hours, pulmonary embolism, unplanned intubation, stroke, deep vein thrombosis, transfusion of packed red blood cells within 72 hours of surgery start time, and sepsis. Cumulative medical complications included all of the aforementioned complications, and cumulative pulmonary complications included pneumonia, on a ventilator for greater than 48 hours, pulmonary embolism, and unplanned intubation. Other complications included cumulative morbidity (consisting of cumulative wound complications and cumulative medical complications), length of stay greater than 1 week, and unplanned readmission.

### Statistical Analyses

Frequencies of demographic, comorbidity, and 30‐day complication variables were calculated for the cohorts of patients with ENT or plastic surgery as the specialty of the attending surgeon. Univariate analysis was performed using Pearson's chi‐square and Fisher's exact test where appropriate, to compare surgical specialty by demographic, comorbidity, and 30‐day complication variables.^16^ When Pearson's chi‐square test showed that there was an independent association between the ordinal variable and the surgical specialty, standardized residuals were calculated such that values greater than 1.96 or less than −1.96 indicate that those specific levels within the ordinal variable significantly differed between the two cohorts.^17^ Backwards stepwise logistic regression models were created for each of those 30‐day complication variables that significantly differed between the plastics and ENT cohorts on univariate analysis. All demographic and comorbidity variables were inputted as independent variables in the backwards stepwise logistic regression models for which the dependent variable was the 30‐day complication variable such that the variables with the greatest *P*‐values (an indicator of weak association with the dependent variable) were iteratively removed until only the independent variables with *P* < .05 remained. Thus, multivariable binary logistic regression was used to determine the independent impact of specialty on outcomes by controlling for demographic and comorbidity variables, with statistical significance defined as a *P*‐value less than .05. Multicollinearity was assessed using multiple linear regression models where the dependent variable was iteratively established as one of the independent variables included in the binary logistic regression model and the dependent variables in the linear regression models were established as the remaining independent variables included in the binary logistic regression model.^18^ Variance inflation factors (VIFs) were calculated for each of the linear regression models such that a value less than 5 indicated a lack of multicollinearity.^19^ Statistical analysis was performed using IBM SPSS Statistics version 25.

## Results

We queried 2127 cases of patients undergoing rhinoplasties. Cases were stratified by specialty into the following two groups: plastics (n = 874 [41.1%]) and ENT (n = 1253 [58.9%]).

On univariate analysis, we found that patients undergoing rhinoplasties by otolaryngologists were more likely to be male as compared to those undergoing this surgery by plastic surgeons (55.8% vs 46.7%, *P* < .001). Additionally, we found that patients in the plastics cohort tended to have more patients of ASA class I (19.2% vs 13.0%, *P* < .001; standardized residuals −2.26, +2.70) compared to those in the ENT cohort. Regarding comorbidities, ENT patients had a higher incidence rate of smoking compared to plastics patients (19.2% vs 14.4%, *P* = .004) (Table 1). There was not a significant difference between cohorts for other comorbidities including diabetes, dyspnea, COPD, hypertension, open wound, bleeding disorders, obesity, steroid use, congestive heart failure, dialysis, disseminated cancer, and greater than 10% loss of body weight in the preceding 6 months. ENT patients had a greater proportion of prolonged operative time compared to plastics patients (59.9% vs 44.4%, *P* < .001) (Table 1).

**Table 1:** Cohort Demographics and Comorbidities

	Plastics (n = 874)	ENT (n = 1253)	*P*‐value
Age cohorts in years, N (%)			.138
≤40	284 (32.5%)	404 (32.2%)	
41‐60	233 (26.7%)	380 (30.3%)	
≥61	357 (40.8%)	469 (37.4%)	
Sex, N (%)			**<.001**
Female	466 (53.3%)	554 (44.2%)	
Male	408 (46.7%)	699 (55.8%)	
Race cohorts, N (%)			.534
White	701 (80.2%)	1025 (81.8%)	
Asian	10 (1.1%)	20 (1.6%)	
Black	43 (4.9%)	53 (4.2%)	
Other	120 (13.7%)	155 (12.4%)	
ASA class, N (%)			**<.001**
I	168 (19.2%)	163 (13.0%)	
II	424 (48.5%)	629 (50.2%)	
III	263 (30.1%)	420 (33.5%)	
IV	19 (2.2%)	41 (3.3%)	
Comorbidities, N (%)			
Smoking	126 (14.4%)	241 (19.2%)	**.004**
Diabetes	72 (8.2%)	120 (9.6%)	.289
Dyspnea	38 (4.3%)	48 (3.8%)	.551
COPD	27 (3.1%)	37 (3.0%)	.856
HTN	291 (33.3%)	421 (33.6%)	.884
Open wound	87 (10.0%)	97 (7.7%)	.074
Bleeding disorder	17 (1.9%)	24 (1.9%)	.961
Obesity	240 (27.5%)	373 (29.8%)	.247
Steroid use	32 (2.5%)	38 (3.0%)	.480
Congestive heart failure	3 (0.3%)	4 (0.3%)	.924
Dialysis	3 (0.3%)	6 (0.5%)	.635
Disseminated cancer	5 (0.6%)	15 (1.2%)	.142
Unexpected weight loss	6 (0.7%)	7 (0.6%)	.710
Prolonged operative time (>155 min)	388 (44.4%)	751 (59.9%)	**<.001**

Bold values denote statistically significance.

Abbreviations: ASA, American Society of Anesthesiology; COPD, chronic obstructive pulmonary disease; ENT, ear, nose, and throat; HTN, hypertension; min, minutes.

On univariate analysis, there were no significant differences between the cohorts for most surgical and medical complications. However, plastic surgery patients were more likely to have sepsis as a postoperative complication compared to ENT patients (0.8% vs 0.1%, *P* = .008) (Table 2).

**Table 2:** Incidence of Complications

	Plastics (n = 874)	ENT (n = 1253)	*P*‐value
Wound complications, N (%)			
Cumulative	24 (2.7%)	43 (3.4%)	.373
Superficial surgical site infection	13 (1.5%)	24 (1.9%)	.458
Deep surgical site infection	4 (0.5%)	7 (0.6%)	.749
Organ/space surgical site infection	4 (0.5%)	2 (0.2%)	.202
Wound dehiscence	5 (0.6%)	12 (1.0%)	.326
Medical complications, N (%)			
Cumulative medical complications	16 (1.8%)	30 (2.4%)	.379
Cumulative pulmonary complications	3 (0.3%)	12 (1.0%)	.096
Pneumonia	1 (0.1%)	5 (0.4%)	.223
Ventilation >48 h	1 (0.1%)	5 (0.4%)	.223
Pulmonary embolism	1 (0.1%)	2 (0.2%)	.785
Unplanned intubation	0 (0.0%)	3 (0.2%)	.148
Stroke	0 (0.0%)	2 (0.2%)	.237
DVT	0 (0.0%)	2 (0.2%)	.237
Transfusion	9 (1.0%)	17 (1.4%)	.500
Sepsis	7 (0.8%)	1 (0.1%)	**.008**
Other, N (%)			
Cumulative morbidity	39 (4.5%)	71 (5.7%)	.217
>1 wk LOS	31 (3.5%)	57 (4.5%)	.254
Readmission	25 (3.5%)	53 (5.3%)	.069

Bold values denote statistically significance.

Abbreviations: DVT, deep vein thrombosis; ENT, ear, nose, and throat; h, hours; LOS, length of stay.

On multivariable analysis, plastics patients had greater odds of acquiring postoperative sepsis as compared to ENT patients (odds ratio [OR]: 23.716, 95% CI: 2.025‐277.764, *P* = .012, Nagelkerke *R* ^2^ = 0.409) and patients with greater than 10% loss of body weight in the preceding 6 months had greater odds of acquiring postoperative sepsis than those who did not have weight loss (OR: 68.121, 95% CI: 7.577‐612.434, *P* < .001) (Table 3). VIFs were less than 1.006 for each of the independent variables in the multivariable analysis.

**Table 3:** Factors Associated With Development of Postoperative Sepsis in Multivariable Analysis

	OR	Significance	95% CI
ENT (reference)			
Plastics	**23.716**	**0.012**	**2.025‐277.764**
Female (reference)			
Male	0.248	0.109	0.045‐1.365
Nonobese (reference)			
Obese	0.095	0.105	0.006‐1.634
No open wound (reference)			
Open wound	4.458	0.062	0.929‐21.391
No unexpected weight loss (reference)			
Unexpected weight loss	**68.121**	**<0.001**	**7.577‐612.434**

Bold values denote statistically significance.

Abbreviations: CI, confidence interval; ENT, ear, nose, and throat; OR, odds ratio.

## Discussion

In this multi‐year retrospective cohort analysis, we aimed to determine differences in demographics, comorbidities, and 30‐day complications in patients undergoing rhinoplasty performed by otolaryngologists versus plastic surgeons. Prior studies comparing outcomes between plastic surgeons and otolaryngologists have had ambivalent findings, including patient‐reported outcomes for rhytidectomy attenuated by geographic location, lack of difference in medical and surgical complications for pediatric rhinoplasties, and fewer complications or no difference in adverse event rate for free flap procedures depending on the study population.^10^, ^15^, ^20^, ^21^, ^22^, ^23^ To the authors' knowledge, this is the largest and only national analysis of adult rhinoplasties based on surgical specialty. This investigation found that despite differences in the demographics and comorbidities of patients undergoing hospital‐based rhinoplasty between the ENT and plastics cohorts, there are no clinically significant differences in the independent odds of 30‐day postoperative complications. These findings are consistent with the results of other studies, such as the retrospective pediatric rhinoplasty cohort studied by Doval et al in 2021.^10^ Ultimately, these results can inform clinical decision‐making by referring providers when choosing which specialty to refer to, highlight differences in patient populations between the two specialties, and increase patient confidence, a key metric that affects patient satisfaction with procedure outcomes, in the surgeon performing rhinoplasty irrespective of specialty.^24^

Our study found that patients with rhinoplasty performed by plastic surgeons tended to be female compared to the greater proportion of males undergoing rhinoplasty by otolaryngologists. Of note, one single‐center two‐time‐comparison study published in 2017 by Loghmani et al showed that female patients were around three times as likely as males to undergo elective, or esthetic, rhinoplasty.^25^ Therefore, the plastics cohort in our study may have had a lower proportion of males due to the greater proportion of esthetic rhinoplasties performed by plastic surgeons. Similarly, the greater proportions of smokers and higher ASA class in the ENT cohort may possibly be due to these patients having higher rates of head and neck cancer and nasal airflow obstruction secondary to pathologies such as obstructive sleep apnea (OSA). These conditions would necessitate functional as opposed to esthetic rhinoplasty. Additionally, although plastic surgeons perform a higher proportion of secondary rhinoplasties, these revision procedures are more likely to be performed because of esthetic concerns than the possible greater proportion of functional secondary rhinoplasties in the otolaryngologists' patient population.^26^, ^27^ Similar to the findings in the pediatric rhinoplasty study, the prolonged operative time in the ENT cohort may be due to a greater proportion of respiratory comorbidities or symptoms.^11^

There was a statistically significant greater incidence and odds of sepsis within 30 days postoperatively in the plastics cohort that persists even in our multivariable analysis. It is not possible to identify the etiology of sepsis, such as surgical site contamination or immunocompromised state, given the limited variables in the NSQIP database. It was found that otolaryngologists perform 71% of rhinoplasties among Medicare beneficiaries, and since Medicare only reimburses functional but not cosmetic rhinoplasty, it may be that the ENT cohort had more functional rhinoplasty than the plastics cohort.^28^ The most common location of nasal obstruction prompting functional rhinoplasty is the internal nasal valve, which can be ameliorated using a spreader graft, usually derived from septal cartilage.^29^ Cosmetic rhinoplasty, on the other hand, may tend to use costal cartilage for ethnic rhinoplasty and alloplastic implants for augmentation rhinoplasty.^30^, ^31^, ^32^ The increased likelihood of multiple sites for cartilage grafts and the use of alloplastic implants may both contribute to the increased odds of postoperative sepsis in the plastics cohort. Although smoking status and prolonged operative length, both of which were present in higher proportions in the ENT cohort, are risk factors for post‐rhinoplasty infection and subsequent sepsis, female sex has also been found to be a risk factor for post‐rhinoplasty infection.^33^, ^34^, ^35^ Our study found that the plastics cohort had a greater proportion of female patients, who may be at increased risk for postoperative infection because of the lower collagen density of the nasal skin and increased proportion of complex augmentation procedures involving materials such as fibrosis‐inducing silicone.^35^, ^36^ However, there were only seven patients in the plastic surgery cohort and one patient in the ENT cohort who had postoperative sepsis, so the sample size of this complication is likely too small to make any clinically meaningful conclusions about differences in short‐term infectious complication rates by surgical specialty.

Prior studies for other procedures have seen a similar lack of differences in overall complication rates between otolaryngologists and plastic surgeons. For example, Jubbal et al found in a national analysis on cleft lip/palate repairs that there were no significant differences in medical or surgical complications between the two specialties.^11^ Additionally, a retrospective cohort analysis of patients from a single level 1 trauma center by Christian et al showed that management of facial fractures yielded no difference in outcomes whether treated by ENT or plastics.^37^

There are several limitations to this study that are important to recognize. Since the inclusion criteria for this study were patients with primary, other, or concurrent procedures with CPT codes for rhinoplasty, some patients may have undergone multiple procedures, which may affect complication rates. The data for the NSQIP database are obtained from more than 700 public and private hospitals throughout the country, but many rhinoplasties are performed in smaller ambulatory surgical centers and private practice offices, so the conclusions of this study may not necessarily be generalized beyond patients undergoing hospital‐based rhinoplasties. Of note, fellowship training of the surgeon performing the rhinoplasty is not included in this analysis, as the “Surgical Specialty” variable in the NSQIP database does not offer this distinction. The findings of this study may be further modified by fellowship‐trained craniofacial plastic surgeons or fellowship‐trained facial plastics and reconstructive otolaryngologists. Additionally, this study did not distinguish between cosmetic versus functional rhinoplasties because CPT codes only describe the procedure that was performed but not the indication for doing so. Though we have presumed a greater proportion of esthetic rhinoplasties being performed in the plastic surgery rhinoplasty cohort based on the findings of other studies, knowing the indications for the rhinoplasties would lend additional statistical credence to the observed differences in gender and ASA class. Despite the inclusion of more than 200 variables, the NSQIP database lacks information on validated patient‐reported esthetic outcomes, comorbidities such as OSA and cocaine use that are established risk factors for complications in rhinoplasty, specificity of reported comorbidities that are important for surgical risk stratification such as type of diabetes and level of glycemic control, and complications beyond 30 days which precludes assessment of rhinoplasty outcomes after the healing process is completed.^1^, ^38^, ^39^, ^40^ Patient‐reported outcome measures and clinician‐assessed outcomes, both of key importance for evaluating the cosmetic and functional success of a rhinoplasty, at 1 year postoperatively or later, were not measured in this study.^41^ The other limitations for this study are true of all retrospective analyses that utilize the ACS‐NSQIP: self‐reported data may be incomplete, coding practices may vary from hospital to hospital, and a causal relationship between surgical specialty and rhinoplasty outcomes cannot be established due to the database's retrospective nature.

Strengths of this study include its large sample size, its inclusion of national data, extensive postoperative complication variables, and multivariable analysis to adjust for potential confounders. Future studies might explore the effects of differences in functional versus cosmetic rhinoplasties on outcomes, which may be present as a variable in databases aside from NSQIP. Prospective cohort analyses in single or multicenter trials that explore rhinoplasty complication rates between ENT and plastics may expand more on our study and better establish causality.

## Conclusion

In this study, we aimed to characterize the associations between surgical specialty and rhinoplasty complications recorded within 30 days postoperatively on a national level. Overall, there was no statistically significant difference between the cohorts for the vast majority of 30‐day postoperative complications. Specifically, however, we found that ENT rhinoplasty patients were more likely to be male, have higher ASA class, smoke, have longer operative time, and have sepsis less often when compared to plastics rhinoplasty patients. Future research that explores the effects of functional versus cosmetic rhinoplasties on these findings can help elucidate the observed significant results.

## Author Contributions

**Sree Chinta**, Conceptualization; design; data collection; data analysis and interpretation; writing; presentation. **Keshav D. Kumar**, Conceptualization; design; data analysis and interpretation; writing. **Dhiraj Sibala**, Conceptualization; design; data analysis and interpretation; writing. **Afash Haleem**, Design; data analysis and interpretation; writing. **Akshay K. Warrier**, Design; writing—review and editing. **Joseph A. Celidonio**, Design; writing—review and editing. **Jean Anderson Eloy**, Design; writing—review and editing; project supervision.

## Disclosures

### Competing interests

The authors report no conflicts of interest.

### Funding source

This work was supported by the Department of Otolaryngology at New Jersey Medical School.
